# Variation in the Mechanical Unfolding Pathway of p53DBD Induced by Interaction with p53 N-Terminal Region or DNA

**DOI:** 10.1371/journal.pone.0049003

**Published:** 2012-11-08

**Authors:** Yukinori Taniguchi, Masaru Kawakami

**Affiliations:** School of Materials Science, Japan Advanced Institute of Science and Technology (JAIST), Nomi, Ishikawa, Japan; Consejo Superior de Investigaciones Cientificas, Spain

## Abstract

The tumor suppressor p53 plays a crucial role in the cell cycle checkpoints, DNA repair, and apoptosis. p53 consists of a natively unfolded N-terminal region (NTR), central DNA binding domain (DBD), C-terminal tetramerization domain, and regulatory region. In this paper, the interactions between the DBD and the NTR, and between the DBD and DNA were investigated by measuring changes in the mechanical unfolding trajectory of the DBD using atomic force microscopy (AFM)-based single molecule force spectroscopy. In the absence of DNA, the DBD (94–293, 200 amino acids (AA)) showed two different mechanical unfolding patterns. One indicated the existence of an unfolding intermediate consisting of approximately 60 AA, and the other showed a 100 AA intermediate. The DBD with the NTR did not show such unfolding patterns, but heterogeneous unfolding force peaks were observed. Of the heterogeneous patterns, we observed a high frequency of force peaks indicating the unfolding of a domain consisting of 220 AA, which is apparently larger than that of a sole DBD. This observation implies that a part of NTR binds to the DBD, and the mechanical unfolding happens not solely on the DBD but also accompanying a part of NTR. When DNA is bound, the mechanical unfolding trajectory of p53NTR+DBD showed a different pattern from that without DNA. The pattern was similar to that of the DBD alone, but two consecutive unfolding force peaks corresponding to 60 and 100 AA sub-domains were observed. These results indicate that interactions with the NTR or DNA alter the mechanical stability of DBD and result in drastic changes in the mechanical unfolding trajectory of the DBD.

## Introduction

**Figure 1:**
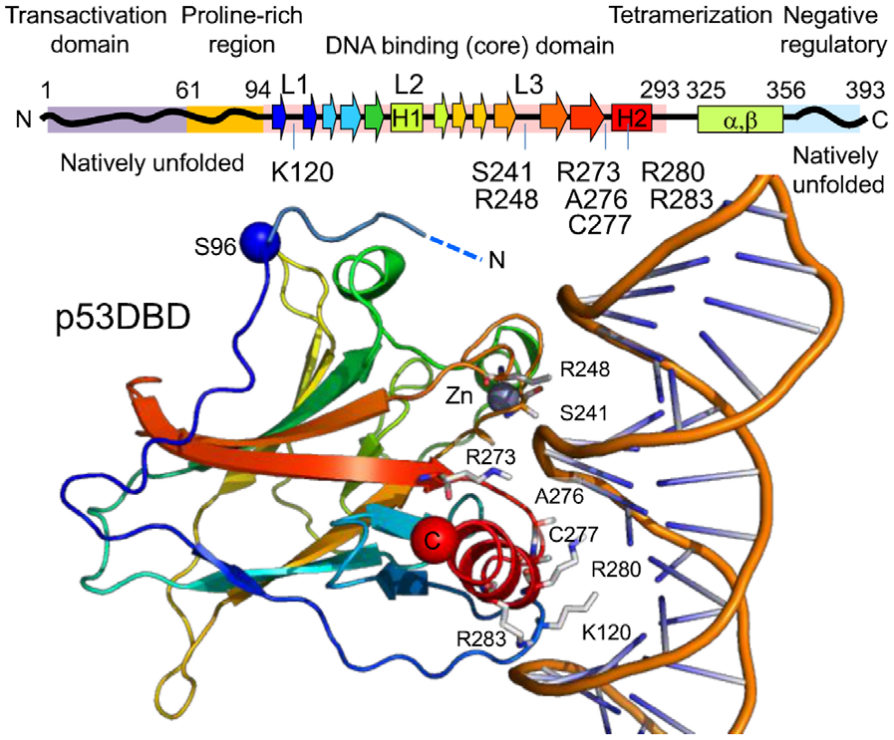
Domain structure of human p53. The domain boundaries follow Joerger and Fersht 2008 (2). The residues that form hydrogen bonds directly with DNA are shown (9). The lower portion is the crystal structure of p53DBD(94–312)-DNA complex, PDB code 1TUP. The N-terminal region (91–95) determined by X-ray crystallography of p53(89–293), 2XWR (7), is aligned and overlaid.

The tumor suppressor protein p53 is a key transcription factor involved in regulation of a variety of cellular processes including the cell cycle, DNA repair, and apoptosis, and more than 50% of human tumors contain a mutation or deletion of the *TP53* gene [1], [2]. p53 functions as a homotetramer. Each chain of p53 consists of two folded domains; the DNA binding domain (DBD), tetramerization domain, and intrinsically disordered regulatory regions (Fig. 1). The N-terminal region (NTR) is intrinsically disordered [3] and contains an acidic transcription-activation domain (TAD) and a proline-rich region (PRR). The TAD plays an important role in regulation of the p53 activity by binding to various partner proteins including MDM2/MDM4 and p300/CBP. The PRR contains PXXP motif and has a tendency to adopt a polyproline II helix structure [4]. The overall flexible structure of the NTR was modeled by combining several approaches [4], [5]. It also has been suggested that a part of the NTR interacts with the DBD [6], [7]. The DBD forms a β-sandwich structure with loops that recognize consensus sequences of DNA. The interaction between the DBD and DNA is essential in function, and has been intensively investigated by several groups [8]–[11].

Atomic force microscopy (AFM)-based single-molecule force spectroscopy (SMFS) is a powerful tool to study not only the dynamics of the mechanical (un)folding of proteins but also protein–ligand interaction at a single-molecule level. There are two strategies to investigate protein–ligand interaction by SMFS. One is the rupture force measurement in which protein and ligand are linked chemically to the AFM stage and cantilever, and the force is applied to break the bonds between protein and ligand [12]. By analyzing the loading rate dependence of the rupture forces (i.e., dynamic force spectroscopy), we can obtain information about the free energy barrier along the mechanical reaction coordinate. Bizzarri et al. have previously reported a study on p53-MDM2 and p53-azurin interactions using such an approach [13], [14]. The other approach is investigating the effect of ligands on the mechanical unfolding of protein [15]–[22]. The binding of a ligand is supposed to induce a change in conformation and/or fluctuation of the host protein which plays an important role in biological functions such as molecular recognition and signal transduction, and therefore is crucial in pharmaceutical research. In this measurement, a tandemly arranged multidomain protein is tethered between the stage and cantilever, and stretching force is applied to unfold domains in the presence/absence of the ligand. Interaction with the ligand possibly affects the mechanical stability of protein that can be assessed by SMFS. Both methods provide unique information about protein–ligand interaction at the single-molecule level.

In this paper, we used the latter approach. We designed two fusion proteins in which p53NTR+DBD(1–293) or p53DBD(94–293) is sandwiched with titin I27 domains. The I27 domains provide a handling region to be picked up by the AFM cantilever and to adhere to the stage, thus the DBD or the NTR+DBD is stretched through its N–C termini. The unfolding of I27 domains provides a characteristic saw-tooth pattern in the force-extension curve, which can be used as a fingerprint of the fusion protein. Using these fusion proteins, we investigated the mechanical unfolding trajectory of the DBD in the absence/presence of the NTR or DNA.

## Materials and Methods

### Construction of Expression Vectors for Fusion Proteins

Expression vectors were constructed from a pentameric I27 polyprotein [(C47S, C63S I27)_5_] using a cassette strategy, as described previously [23], [24]. The third I27 domain was replaced with a PCR-generated cassette encoding full-length human p53 coding region using *BssHII* and *SacI* restriction sites. The p53 cDNA was a gift from Prof. Ichimiya and Prof. Tokino (Sapporo Medical Univ.) [25]. The expression vector for I27-I27-p53NTR+DBD(1–293)-I27-I27 was constructed by deleting the C-terminal region of p53, which was performed by inverse PCR. Similarly, vector coding I27-I27-p53DBD(94–293)-I27-I27 was created by deleting the N-terminal region of p53. DNA sequence analysis confirmed the sequence of the coding region of the fusion proteins. The amino acid sequences were MHHHHHHSS-(I27)-VEAR-(I27)-LIEAR- {p53NTR+DBD(1–293) or p53DBD(94–293)}-LSSAR-(I27)-LIEARA-(I27)-CC.

### Expression and Purification of Fusion Proteins

I27-I27-p53NTR+DBD(1–293)-I27-I27 or I27-I27-p53DBD(94–293)-I27-I27 protein was overexpressed in BLR(DE3)pLysS *E. coli* cells (Merck KGaA, Darmstadt, Germany). Cells were cultured in 800 ml of LB medium at 37°C until they reached an OD_600_ of 0.6. After addition of IPTG to a final concentration of 1 mM, cells were grown at 16°C overnight and then harvested by centrifugation. The cell pellet was resuspended in a buffer (50 mM NaH_2_PO_4_, 300 mM NaCl, 10 mM imidazole, 1% protease inhibitor cocktail set VII (Calbiochem, CA, USA), pH 6.5) and lysed by sonication on ice. The protein was purified by affinity chromatography using Ni-NTA column (Qiagen, CA, USA) according to the manufacturer’s protocol, and by using a Heparin column (GE Healthcare, NJ, USA) with gradient elution (0–2 M NaCl, 10 mM sodium phosphate, pH 7.0). Finally, gel filtration chromatography using Sephacryl S-200 HR column (GE Healthcare, NJ, USA) equilibrated with PBS plus 10 mM DTT was carried out. All purification procedures were performed at 4°C. The purity of the protein was checked by SDS-PAGE analysis.

### AFM Force Spectroscopy

Typically, 1–2 µl of protein stock solution (approximately 3 mg/ml) was added to 200 µl of PBS (140 mM NaCl, 10 mM phosphate buffer, and 3 mM KCl, pH 7.4) plus 10 mM MgCl_2_, 10 mM DTT, and incubated on freshly cleaved mica substrate for 10 min at room temperature. The final protein concentration was 0.2–0.4 µM. For the experiments in the presence of DNA, pre-annealed 16-mer dsDNA oligonucleotides (5′-CCTAGACATGCCTAAT-3′, purchased from Operon Biotechnologies, Tokyo, Japan), which contained one p53 consensus half-site (underlined), was added to the protein stock solution with a molar dsDNA:p53 ratio of 1∶2, 10∶1, or 20∶1. The p53DBD binds to this DNA as a dimer [26]. The fusion proteins were picked by non-specific protein-cantilever and protein-mica surface interaction, and then stretched at a pulling speed of 500 nm/s using a Picoforce AFM with Nanoscope 3D controller (Burker Japan, Tokyo, Japan) controlled by custom-built software operating on Igor Pro 5.05A (Wavemetrics, OR, USA). All measurements were performed using MLCT cantilever (Burker Japan) whose spring constant was estimated to be 60–70 pN/nm from the equipartition theorem [27], [28]. The force-extension curves were analyzed using the worm-like chain (WLC) model [29],
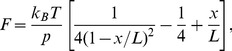
(1)where *F* is the force, *k_B_* is the Boltzmann constant, *T* is the temperature in Kelvin, *p* is the persistence length, *x* is the extension, and *L* is the contour length of the polymer. The persistence length *p* was fixed at 0.6 nm. We selected the force curves of interest by the following criteria: (1) three or four I27 unfolding peaks were found and (2) these contour length and increment of the contour length were consistent with that the expected length.

## Results and Discussion

### Sole p53DBD Unfolds Mechanically via Two Pathways

**Figure 2:**
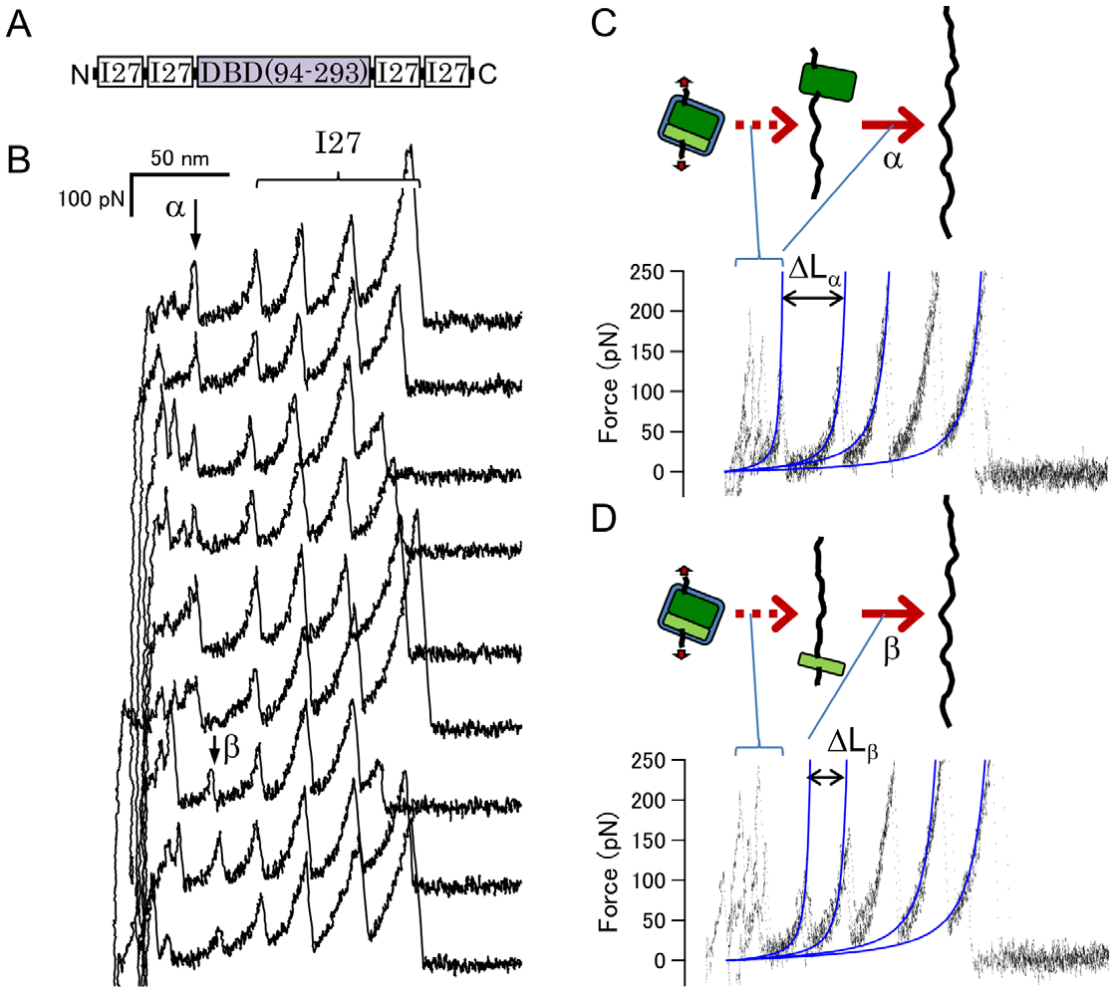
The mechanical unfolding trajectory of p53DBD. (A) The design of p53DBD-I27 fusion protein. (B) Force-extension curves of the fusion protein. All curves are aligned with the first I27 force peak. (C) Superimposed traces that show type 1 intermediate (upper four traces in (B)). Solid lines are fits of the WLC model. The contour length increment (ΔL_α_) is 34±1 nm. (D) Type 2 intermediate found in the lower three traces in (B). The ΔL_β_ is 22±3 nm.

The mechanical unfolding trajectory of p53DBD was investigated by AFM-SMFS. Figure 2 shows the force-extension traces observed in this study, exhibiting a saw-tooth pattern. Each rising phase of these force peaks can be fitted with the WLC model (eq. 1) showing the entropic elasticity of the flexible linkers and unfolded region(s) of the polypeptide chain. The last three or four unfolding force peaks with the contour length increment (ΔL) of around 28 nm are ascribed to the unfolding of the I27 domains within the construct [30], [31]. The other force peaks in the shorter extension region are attributed to the unfolding event of the DBD. Interestingly, at least two patterns of force profiles of the DBD were observed as shown in Fig. 2, Table 1, and Table 2. One is characterized by an unfolding peak with ΔL of 34±1 nm (indicated as α in the upper six traces in Fig. 2B and Fig. 2C). The other shows a distinctive force peak with ΔL of 22±3 nm (β in the lower three traces in Fig. 2B and Fig. 2D). Each peak indicates an unfolding event of a residual structure, which comprises approximately 100 and 60 amino acids, respectively, assuming that the length of a single peptide bond is 0.36 nm [32]. The unfolding force of the former residual structure was 121±17 pN, which is slightly larger than that of the latter, 77±33 pN (Table 2). Unfortunately, an extremely low yield of successful extension of this fusion protein makes it difficult to obtain sufficient amount of data for statistical analysis. However, coexistence of these two unfolding patterns was demonstrated reproducibly and indicated that there are at least two distinct pathways in the mechanical unfolding of the DBD. Intriguingly, complexity has been found in the DBD refolding from a chemically denatured state [33], although the pathway in the chemical (un)folding is not necessarily the same as that in the mechanical unfolding [34], [35]. Both results suggest multidomain/multilobe nature in p53DBD (un)folding.

**Table 1:** The number of force curves observed in this study.

	α	β	γ	β′α′	Others*
p53DBD	4	5	0	0	9
p53NTR+DBD	0	0	9(3)†	0	10
p53NTR+DBDwith DNA	0	0	2(1)†	7	5

**Figure 3:**
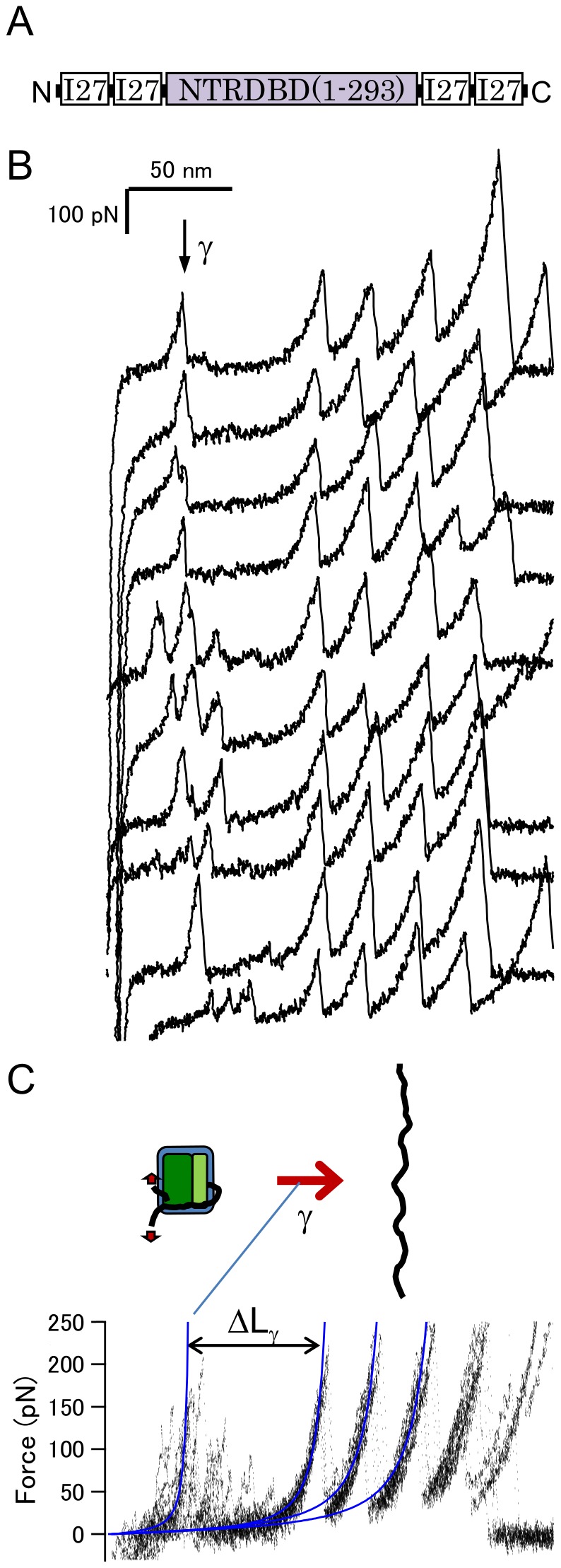
The mechanical unfolding trajectory of p53NTR+DBD. (A) The design of (p53NTR+DBD)-I27 fusion protein. (B) Force-extension curves of the fusion protein. (C) Superimposed traces shown in (B). The unfolding force peak with ΔL_γ_ of 76±3 nm is shown in the upper six traces of (B).

**Figure 4:**
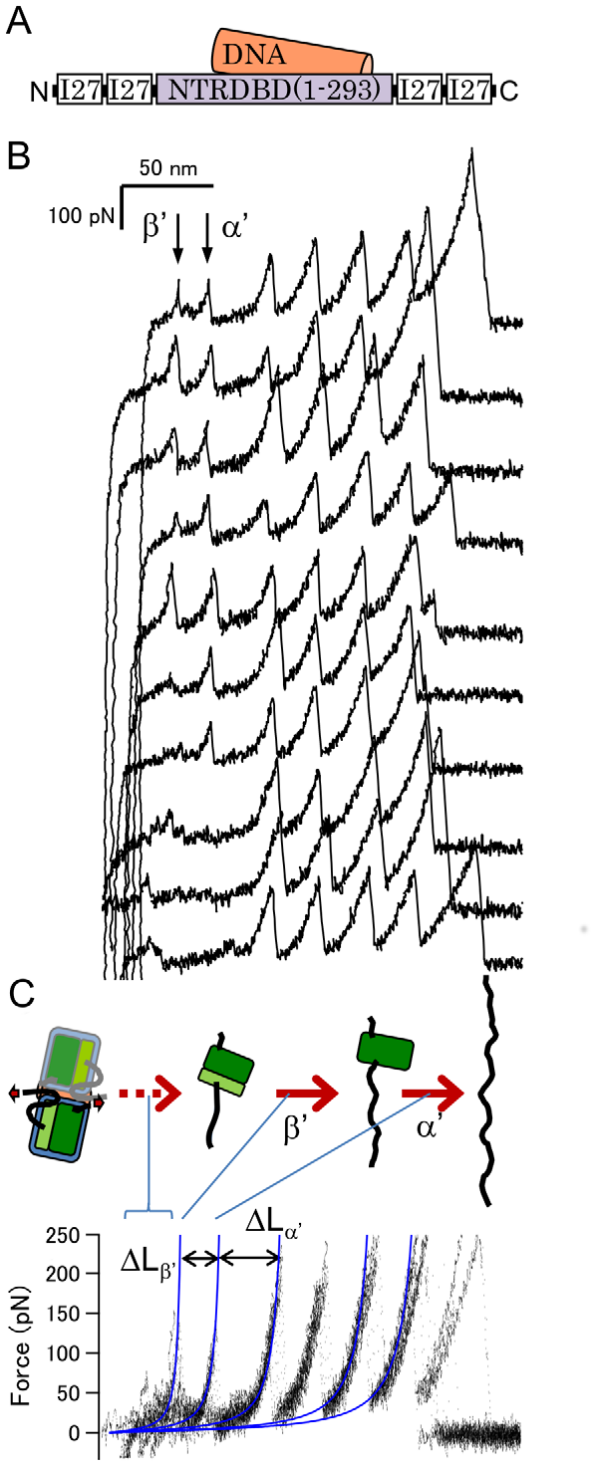
The mechanical unfolding trajectory of p53NTR+DBD in the presence of DNA. (A) A schematic representation of the sample. (B) Force-extension curves under these conditions. (C) Superimposed traces shown in (B). The two unfolding force peaks were shown in the upper seven traces of (B). ΔL_β′_ is 21±3 and ΔL_α′_ is 35±1 nm.

* This category includes the curves in which the first p53 peak is unclear due to surface-cantilever interaction or contaminants (for example, the 5^th^ and 6^th^ curves in Fig. 2B) and curves which cannot be categorised due to poor reproducibility (ex. the bottom three curves in Fig. 3B and 4B).

† The number of curves that showed additional peaks is shown in parenthesis (ex. the 5^th^ and 6^th^ curves in Fig. 3B).

**Table 2:** The unfolding force (F_UN_) and contour length increment (ΔL).

		F_UN_ (pN)	ΔL (nm)
p53DBD	I27	214±49	27±2
	α	121±17	34±1
	β	77±33	22±3
p53NTR+DBD	I27	190±43	28±2
	γ	136±45	76±3‡
p53NTR+DBDwith DNA	I27	192±38	26±2
	α′	110±14	35±1
	β′	89±35	21±3
	γ	74±22	73±2‡

‡ The ΔL is measured from the first peak of I27.

### Mechanical Unfolding of p53NTR+DBD Happens not Solely on the DBD but also Accompanying a Part of the NTR

We investigated the mechanical unfolding trajectory of p53DBD+NTR to reveal the effect of NTR on the mechanical stability of the DBD. Figure 3 shows the force-extension curves of the fusion protein. The NTR+DBD did not show the unfolding patterns found in the case of the DBD alone, but striking heterogeneous unfolding patterns were observed (Fig. 3B and C). Of the heterogeneous patterns, we observed a high frequency of force peaks with ΔL of 76±3 nm (γ in the upper four traces in Fig. 3B). These force peaks indicate an unfolding of a domain of approx. 220 AA, which is apparently larger than that of the DBD alone (199 AA). These results imply that a part of the NTR binds to the DBD, and mechanical unfolding happens not solely on the DBD but accompanying a part of NTR. Furthermore, the absence of force peaks with ΔL of around 20 or 35 nm suggests the mechanical unfolding pathway is completely different from those of the DBD alone. In some of the force curves with multiple peaks, one of the peaks was found at the same position as the γ peak (ΔL of 76 nm measured from the first peak of I27) as shown in the 5^th^ and 6^th^ traces in Fig. 3b. We categorized them into the γ group.

Interaction between the NTR and the DBD has been suggested from single-molecule FRET experiments [6]. It has been shown that the residues 86–93, the hinge region between the DBD and proline rich region interact directly with the DBD; in particular, Trp91 forms a cation-pi interaction with Arg174 [7] (see Fig. 1). Natan et al. pointed out that the hinge region can be considered part of the DBD. Our finding using SMFS, as described above, is consistent with this idea. In the case of our measurement, truncation of this region is supposed to alter the pulling direction. The change in the pulling direction can cause a drastic effect on the apparent mechanical stability and unfolding pathway of the DBD because the mechanical stability of a domain is generally anisotropic [36]–[38].

The hinge region is not essential for the overall structure of the DBD [7]. A recombinant protein encoding residues 102–293 is stable and has DNA binding activity [8]. It is likely that these regions (86–102) fold with low cooperativity and/or conformational fluctuations. If it is the case, a hypothesis may be suggested: these regions may have a heterogeneous structure and/or unravel heterogeneously under an applied force, which causes the observed heterogeneous unfolding patterns.

### Binding to DNA Alters the Mechanical Unfolding Pathway

The effect of DNA binding on the mechanical unfolding trajectory of p53DBD+NTR was investigated. This construct lacks the tetramerization domain of p53, but binds to a consensus half-site DNA as a dimer (10,26). In the presence of the DNA, the mechanical unfolding trajectory of NTR+DBD showed a different pattern to that without DNA (Fig. 4, Table 1 and 2). Two consecutive unfolding force peaks with ΔL of 21±3 and 35±1 nm were observed (β′ and α′, respectively, in the upper seven traces in Fig. 4B). Since the ΔL values and the unfolding forces of each unfolding event are very similar to those found in case of DBD alone (see Table 2), presumably the same residual structures were detected. In contrast to the case of a solo DBD, a single unfolding pathway was dominant. These results suggest that the larger residual structure (ΔL of 35) is particularly stabilized by interaction with DNA. Previous studies have shown that the interaction with DNA does not alter the conformation of the DBD, and several residues form a hydrogen bond with DNA (9) (see Fig. 1). Most of these residues are located near the C-terminal of the DBD, the loop 3, and the vicinity of the helix 2, except for K120. Therefore, such regions near C-terminal are presumably included in the larger residual structure. To obtain more detailed information on these residual structures, mutation studies and/or computer simulation studies are necessary. However, these results indicate that the binding of DNA abrogates the effect of the NTR binding, and alters the mechanical unfolding trajectory of the DBD. The interaction between the NTR and DBD is affected in the presence of DNA presumably by steric hindrance and/or electric repulsion with DNA because of the negative charge of both the TAD and DNA. Previous studies have shown that the TAD binds weakly to the DBD in an electrostatic manner [39].

**Figure 5:**
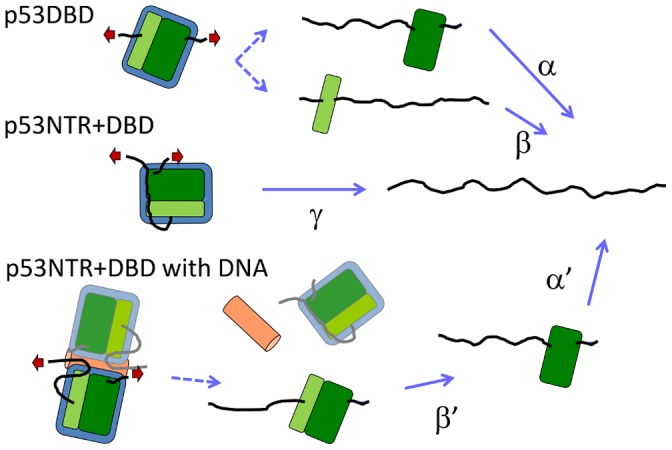
A schematic drawing of the mechanical unfolding pathways of the DBD and the NTR+DBD in the absence and presence of DNA observed in this study.

### Unfolding Scenarios

A schematic mechanical unfolding scenario of the DBD that was reproducibly observed in this study is shown in Fig. 5. The solo DBD mechanically unfolds via at least two pathways, showing the complexity in the mechanical unfolding of the DBD at the single-molecular level. Note that since the number of datasets obtained in this study is not large due to an extremely low yield of successful extension, it is uncertain that all the pathways have been observed and categorized. Coexistence of multiple mechanical (un)folding pathways was recently found in T4 lysozyme [40], calmodulin [41], and maltose binding protein [20] by SMFS, and explained by the kinetic partitioning mechanism. The unfolding pathway of the DBD alters depending on the binding of the NTR or DNA in distinct manners. This drastic change in the unfolding trajectory may be due to the change in the pulling geometry, and also thermodynamic stabilization by interaction with the ligands. Previous studies have shown that ligand binding increases the mechanical stability [15], [17]–[19], [22], which causes an alteration in partitioning of unfolding pathways [20], or produces a new unfolding pathway [21]. In the former two cases, the change of unfolding force or partitioning of pathways needs statistical analysis to be clarified because these processes are essentially stochastic. On the other hand, the appearance of a new unfolding trajectory is easy to detect even in a single force curve. From the results of this study, we propose that the design of a fusion protein, where the ligand changes the pulling direction is a promising method for the effective detection of protein–ligand interaction using SMFS.
